# Invisible Ties: Methamphetamine Use and Eating Disorders in Female Patients Methamphetamine use and eating disorders (EDs) frequently coexist in female patients, with stimulant misuse often serving as a mechanism for appetite suppression

**DOI:** 10.1192/j.eurpsy.2026.10224

**Published:** 2026-07-31

**Authors:** S. H. Elmasry

**Affiliations:** Psychiatry, Ain Shams University, Cairo, Egypt

## Abstract

**Introduction:**

Eating disorders (EDs) and substance use disorders (SUDs) often coexist, leading to significant clinical challenges and poorer treatment outcomes. This comorbidity is characterised by a complex interplay of biological, psychological, and social factors, which complicate both diagnosis and treatment. However, there is a notable gap in research populations, where cultural, social, and economic factors can influence the manifestation and progression of these disorders. The stigma associated with both eating disorders and substance abuse further complicates the clinical landscape. Patients may be reluctant to seek help due to societal judgement and lack of awareness, leading to underdiagnosis and inadequate treatment.

**Objectives:**

- To determine the rate of eating disorders among female patients with methamphetamine substance use disorders.
- To identify the demographic, psychological, and social correlates associated with the co-occurrence of these disorders.

**Methods:**

- **Type of Study: It** is a cross-sectional observational study.

**Age range:** 18-45 years old.

Egyptian nationality

- study tools:
- **MINI:** The Mini-International Neuropsychiatric Interview
- **Addiction severity index (ASI)**

**Eating Attitudes Test-26 (EAT-26)**

- **Dysmorphic Concern Questionnaire (DCQ)**

**Results:**

This study found that many female adolescents who use methamphetamine also have body dysmorphia (BDD), with 35% of those with BDD also having an eating disorder. These include anorexia nervosa (15%), bulimia nervosa (9%), and other eating disorders (22%). Methamphetamine use, often linked to body image improvement and self-esteem, was reported by 25% of women with bulimia and 6% with anorexia. The use of methamphetamine to suppress appetite and enhance body image often worsened disordered eating behaviours. Additionally, 60% of women with eating disorders had a substance use disorder, and 45% of those with a substance use disorder had an eating disorder. This combination of disorders significantly increased the risk of medical and psychiatric complications, including mood disorders and psychosis.

**Conclusions:**

The co-occurrence of methamphetamine use and eating disorders in female adolescents represents a major clinical challenge. Integrated treatment approaches that address both conditions simultaneously are essential for improving outcomes. Clinicians should be aware of the heightened risks and the importance of a comprehensive, trauma-informed treatment plan. The findings highlight the need for further research to refine these approaches and ensure effective, long-term recovery for this vulnerable population.

**Disclosure of Interest:**

None Declared

